# Three-dimensional chromatin landscapes in MLLr AML

**DOI:** 10.1186/s40164-024-00523-5

**Published:** 2024-05-22

**Authors:** Pinpin Sui, Zhihong Wang, Peng Zhang, Feng Pan

**Affiliations:** 1 Department of Cell Systems & Anatomy, University of Texas Health Science Center at San Antonio, San Antonio, TX USA; 2 Department of Molecular Medicine, University of Texas Health Science Center at San Antonio, San Antonio, TX USA; 3 Department of Pathology, Stanford University, Stanford, CA USA

## Abstract

Rearrangements of the mixed lineage leukemia (MLLr) gene are frequently associated with aggressive acute myeloid leukemia (AML). However, the treatment options are limited due to the genomic complexity and dynamics of 3D structure, which regulate oncogene transcription and leukemia development. Here, we carried out an integrative analysis of 3D genome structure, chromatin accessibility, and gene expression in gene-edited MLL-AF9 AML samples. Our data revealed profound MLLr-specific alterations of chromatin accessibility, A/B compartments, topologically associating domains (TAD), and chromatin loops in AML. The local 3D configuration of the AML genome was rewired specifically at loci associated with AML-specific gene expression. Together, we demonstrate that MLL-AF9 fusion disrupts the 3D chromatin landscape, potentially contributing to the dramatic transcriptome remodeling in MLLr AML.

**Supplementary Information:**

The online version contains supplementary material available at 10.1186/s40164-024-00523-5.

**To the editor,**

The clinically important and genetically well-defined MLLr leukemia accounts for up to 50% of infant and 10% of adult acute leukemia that are associated with very poor prognosis and chemo-resistance [1–3]. The current mechanistic understanding of MLLr AML initiation and progression have not yet translated into therapeutic success due to the lack of an accurate and reliable human model, and the complexity of genomic events contributing to disease [4]. Recent CRISPR/Cas9-mediated generation of MLLr in human hematopoietic stem and progenitor cells (HSPCs) has enabled the modeling of key aspects of AML biology [5–7]. To understand how the 3D topology of the genome contributes to the leukemogenesis of MLLr AML, we coupled assay for transposable-accessible chromatin using sequencing (ATAC-seq) and RNA sequencing (RNA-seq) to enhanced high-resolution chromosome conformation capture (Micro-C) in gene-edited MLL-AF9 AML cells (Fig. 1A). Normal human umbilical cord blood CD34 + cells were selected as controls since they are considered to be healthy donor cells of origin for AML. We firstly estimated the similarity between AML samples and HSPCs using principal component analysis (PCA) and differential chromatin accessibility analysis of ATAC-seq profiles (Fig. 1B-C). AML-specific accessible DNA regions exhibited a significant AML signature, indicating that these cells are a suitable experimental system to map the 3D genome architecture of AML (Fig. S1A-D). On average, around 800 million paired-end reads were generated in each Micro-C library. Unsupervised hierarchical clustering using Micro-C matrices clearly separated HSPCs and leukemia cells (Fig. S2A), indicating a specific chromatin structural landscape of MLLr AML. Notably, there were substantial changes in compartmentalization in leukemia cells vs. in HSPCs (Fig. 1D-E and Fig. S2B). About 1,476 A-to-B compartment switch and 2,251 B-to-A switch were observed when comparing AML samples with HSPCs at 100 kb resolution (Fig. 1D). Moreover, genes in the A-to-B switching regions had decreased expression, (Fig. 1F-G). To further explore the chromatin structure of MLL-AF9 AML, we investigated differences in TAD number and strength between AML cells and HSPCs at 25 kb resolution. Here, we observed that AML cells exhibited a slightly lower number of TADs with weaker strength compared to HSPCs. (Fig. 1H-I and Fig. S2C-F).

**Fig. 1:**
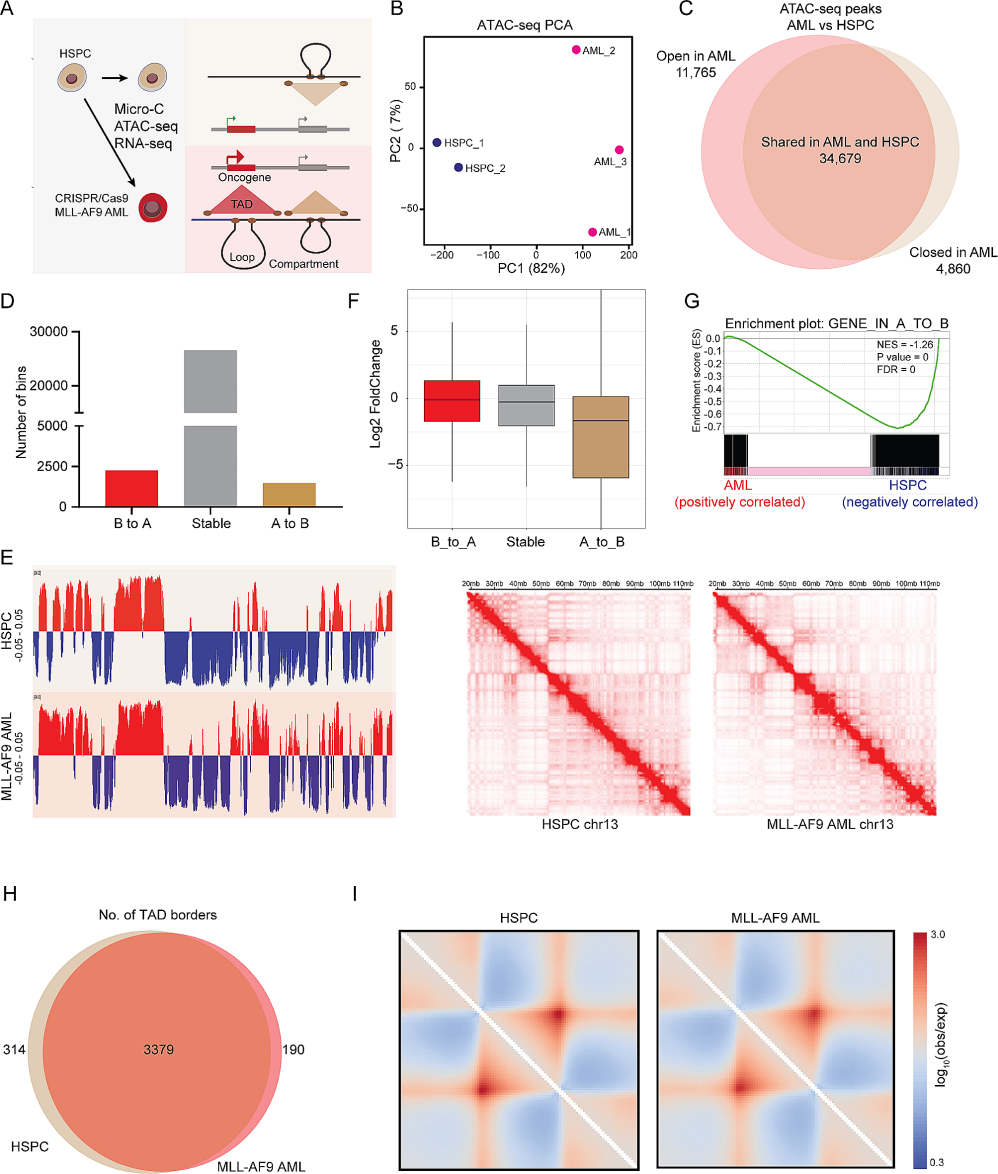
Large-scale 3D genome organization in MLL-AF9 AML. (**A**) Schematic overview of the 3D genome characterization of MLL-AF9 AML cells generated through CRISPR/Cas9-mediated gene editing. (**B**) PCA of chromatin accessibility profiles of HSPCs and gene-edited MLL-AF9 AML cells. (**C**) Venn diagram representation of the differential chromatin accessibility between ATAC-seq peaks of HSPC and MLL-AF9 AML. (**D**) Number of **A**/B compartment switching in MLL-AF9 AML samples compared with HSPCs. (**E**) PCA analysis for the first principal component representing compartment A and B at 100 kb resolution and representative Micro-C interaction matrix on chr13. (**F**) Gene expression alterations associated with changes in the A/B compartment. (**G**) GSEA enrichment plots showing that downregulated genes are enriched in A to B switch. (**H**) Overlap of HSPC TADs and MLL-AF9 AML TADs. (**I**) Aggregate domain analysis (ADA) showing all TAD in HSPC (left) and MLL-AF9 AML (right)

Chromatin loops were detected using Mustache at 10 kb resolution [8]. This identified 5,731 distinct healthy donor-specific loops and 2,679 AML-specific loops (Fig. 2A). The MLL-AF9 AML-specific loops also showed subtype-specific patterns and contained many known MLL target genes, such as *UBE2J1*, *PARP8*, and *PHC2*, and non-coding elements in the loop anchors (Fig. 2B-D and Fig. S3A-B). Notably, we did not observe significant loop alterations involving the well-known MLL-AF9 signature genes *HOXA9* and *MEIS1* (Fig. S3B). This suggests that the 3D genome dynamics in disease pathogenesis may be more complex than previously understood, and potentially independent of the HOXA9/MEIS1 axis in MLLr AML. Similarly, the HSPC-specific loops contained HSPC signature genes (*KIT*, *NRIP1*, and *IGF1R*). The MLL-AF9 AML-associated loops demonstrated enrichment for several key transcription factor (TF) motifs suggesting an interactive TF network in MLL-AF9 AML (Fig. 2E). The motifs constituted binding sites for TFs involved in hematopoietic lineage specification (PU.1) and MLL leukemia pathogenesis (IKZF1 and MYB). Recent studies indicate that chromatin loops that connect promoters to enhancers and silencers are essential for gene regulation [9, 10]. In support of this notion, we noticed 597 genes were significantly upregulated and 1368 genes were downregulated in dysregulated AML loops (Fig. S3C-F).

**Fig. 2:**
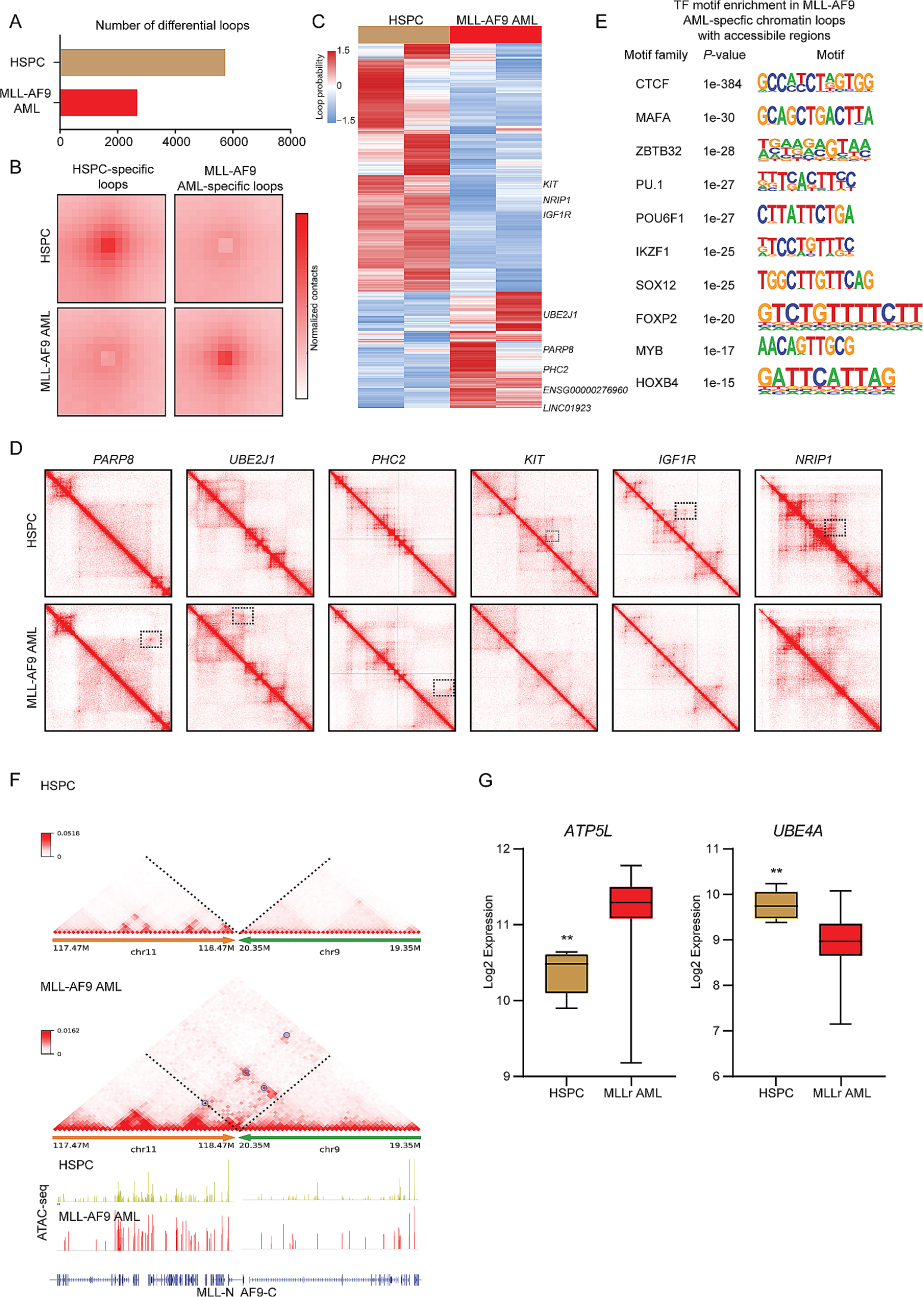
MLL-AF9 AML specific chromatin loops. (**A**) Number of HSPC- and MLL-AF9 AML-specific loops using the mustache at 10 kb resolution. (**B**) APA plot for MLL-AF9 AML interaction-increased and interaction-decreased loops compared with HSPC. (**C**) Heatmap shows the subtype-specific loop analysis for HSPCs and MLL-AF9 AML samples. Each row is a loop and the values are the scaled interaction frequency extracted by Juicer (Observed/Expected). (**D**) Micro-C interaction matrix surrounding HSPC- and MLL-AF9 AML-specific genes. (**E**) De novo motif analysis showing the enrichment of transcription factors in the anchor regions associated with open chromatin regions of MLL-AF9 AML-specific chromatin loops using HOMER. (**F**) Enhancer-hijacking and silencer-hijacking events analyzed by NeoLoopFinder (black circles). (**G**) Box plot of ATP5L and UBE4A expression levels in normal HSPCs and MLLr AML cells from BloodSpot [12]

The observed AML bearing MLL-AF9 translocation raised the possibility that the structural variation can disrupt three-dimensional genome organization and induce enhancer/silencer hijacking. To assess potential chromatin interactions induced by translocation, the NeoLoopFinder tool was used to identify genes associated with enhancer/silencer hijacking [11]. A representative example is shown in Fig. 2F, which shows the fusion between chromosome 11 and chromosome 9 in AML but not in HSPC. This neo-loop connected *ATP5L* (located on chromosome 11) to several putative enhancers on chromosome 9. By contrast, no such inter-chromosomal Micro-C signals were observed in HSPC. As expected, *ATP5L* showed increased expression in MLLr AML samples that exhibited enhancer-hijacking. However, *UBE4A* showed decreased expression in MLLr AML samples, suggesting silencer-hijacking events were identified in MLLr AML samples (Fig. 2G and Fig. S3G).

Together, the observed 3D genome alterations, including compartmentalization changes and MLL-AF9 specific loops, resonate with similar recent findings [10], further supporting the role of 3D genome dysregulation in MLLr AML progression. However, considering the limitations of sample size and loop validation, an interesting future experiment would be to incorporate ChIP-seq for CTCF, H3K27ac, and H3K27me3 to systematically separate loop-associated enhancers and silencers and further validate the identified AML-specific loops. This additional data would enhance our understanding of the specific mechanisms by which MLL-AF9 alters the 3D chromatin landscape and contributes to leukemogenesis.

### Electronic supplementary material

Below is the link to the electronic supplementary material.

Supplementary Material 1

## Data Availability

The raw data of RNA-Seq and Micro-C reported in this paper have been deposited in the Gene Expression Omnibus database (accession number GSE244472).

## Abbreviations

3D: Three-dimensional
AML: Acute myeloid leukemia
HSPC: Hematopoietic stem and progenitor cells
Micro-C: An enhanced 3 C-based technique using Micrococcal nuclease
MLLr: Rearrangements of the mixed lineage leukemia
TAD: Topologically associating domain
TF: Transcription factor
