# Supplementary material for: Three-dimensional chromatin landscapes in MLLr AML
Source: Exp Hematol Oncol. 2024 May 22;13:56. doi: 10.1186/s40164-024-00523-5 (PMC11110396; doi:10.1186/s40164-024-00523-5)
Supplement: Supplementary file 1 — Supplementary Material 1 [file 40164_2024_523_MOESM1_ESM.docx]

**Supplementary data**

**Materials and methods**

**Cell culture**

Human CD34^+^ HSPCs and CRISPR-Cas9 engineered MLL-AF9 leukemia cells were cultured in StemSpan SFEM II medium with SCF (50 ng/mL), thrombopoietin (100 ng/mL), Flt3 ligand (100 ng/mL), IL-6 (100 ng/mL), IL-3 (50 ng/mL), G-CSF (50 ng/mL), UM729 (0.75 µM), StemRegenin 1 (0.75 µM), and 20% FBS at 37°C, 5% CO_2_ as previously described [1].

**Micro-C library preparation and data analysis**

Micro-C libraries were performed following Dovetail Micro-C Kit protocol (Cat# 21006, Dovetail Genomics, Scotts Valley, CA, USA). Briefly, 1x10^6^ leukemia and healthy donor cells were resuspended in 1xPBS and crosslinked with 3mM DSG and 1% formaldehyde. Treated cells were then washed and digested with 0.5 uL MNase in 100 uL of Nuclease digest buffer. Digested chromatin was thawed by addition of 3µl of 20% SDS and capture on chromatin capture beads. Chromatin bound DNA fragments were end repaired and A-tailed using the End Polishing enzyme mix and buffer. Dovetail bridge adapters were ligated to the free ends of chromatin-bound DNA fragments, following the Micro-C kit protocol. This was followed by intra-aggregate ligation to form chromatin-chromatin long-range interactions. Finally, the chromatin-bound DNA was recovered by treating the sample with proteinase K to reverse the crosslinks, and then purifying the DNA using SPRIselect beads (Beckman Coulter). After DNA purification, PCR was performed to amplify the library and size range between 350–1,000 bp was selected for the library. The final libraries were sequenced by Illumina NovaSeq 6000 with the mode of paired-end 150bp. Trim galore (v 0.6.7) (https://github.com/FelixKrueger/TrimGalore) was used to trim the raw sequencing data with the parameter of –paired -q 25 –phred33 –length 35 -e 0.1 –stringency 2. Clean reads from each library were aligned to the human genome (GRCh38) using BWA (v 0.7.17-r1188) [2]. Pairtools (v1.0.2) was used to process the alignment output and generate the final bam file used for the contact matrix [3]. Juicer (v 1.22.01) and cooler (v 0.9.1) were used to generate .hic and .cool file, respectively [4, 5]. A/B compartments were identified by FAN-C (v 0.9.25) at 100 kb resolution [6]. TADs were identified by juicer arrowhead (v 1.22.01) at 25 kb resolution. Loops were identified by mustache (v 1.3.1) at 10 kb resolution [7]. Insulation scores were calculated by cooltools (v 0.5.4) at 25 kb resolution (https://doi.org/10.1101/2022.10.31.514564). Aggregate domain analysis (ADA) was performed with coolpup.py (v 1.1.0) at 25 kb resolution [8]. The overlapped regions between AML-specific chromatin loop anchors (both left and right, 10kb each) and ATAC-seq peaks were subject to HOMER's findMotifsGenome.pl tool for motif discovery.

**ATAC-seq analysis**

ATAC-seq was performed as described [9, 10]. Briefly, healthy donor and AML cells were lysed in cold lysis buffer (10 mM Tris-HCl, pH 7.4, 10 mM NaCl, 3 mM MgCl2, and 0.1% IGEPAL CA-630). Nuclei were centrifuged at 500 × g for 10 min, 4°C. Nuclei extract were then incubated with Nextera Tn5 Transposase, 2× TD buffer, and nuclease free water at 37°C for 30 min with gentle mixing. After DNA purification with the MinElute PCR Purification Kit (Qiagen), PCR was performed to amplify the library for additional cycles distinctively according to a quantitative PCR reaction for optimum cycles. The final libraries were sequenced by Novogene. Adapter sequences were trimmed and reads were mapped to Hg38 using Bowtie2 [11]. ATAC-seq peak calling was performed with Genrich (https://github.com/jsh58/Genrich) with default setting. HOMER's getDifferentialPeaks tool was used to identify differentially accessible peaks between MLL and HSPC samples. Peaks were assigned to genes based on GREAT analysis (<https://great.stanford.edu/great/public/html/>). Bias corrected bigwig files were generated by TOBIAS for genomic track visualization [12]. The list of genes associated with differentially accessible peaks was uploaded to Enrichr (<https://maayanlab.cloud/Enrichr/>) and GO terms related to biological processes and diseases were analyzed (p-value = 0.05). ATAC-seq datasets are available from GEO under accession number GSE228237.

**RNA-seq analysis**

RNA was extracted from control and leukemia cells with RNeasy Plus Mini Kit (Qiagen) according to the manufacturer's instructions. RNA-seq libraries were generated and sequenced by Illumina NovaSeq 6000 (2x150bp). Trim galore (v 0.6.7) was used to trim the raw data with the parameter of --pair -q 25 --phred33 --length 35 -e 0.1 --stringency 2 (https://github.com/FelixKrueger/TrimGalore). The clean reads were aligned to the human genome (GRCh38) using STAR (v 2.7.10a) [13] and then HTSeq (v 2.0.2) [14] was used to calculate the raw read counts for each gene and each sample. The count matrix was used to identify differentially expressed genes (DEGs) by DESeq2 (v 1.36.0) [15] with the cutoff of FDR < 0.05 and |Fold change| >2.

**Linking Micro-C, ATAC-seq and RNA-seq data**

Differential expression analysis was performed on the entire set of genes followed by examining the overlap between differentially expressed genes (DEGs) and genes located within differentially altered TADs and loops, and open chromatin regions, respectively. Log-transformed gene expression fold changes were used to compare the overall expression change trends of genes located within different chromatin-changed groups, such as A/B compartment stable and switched regions.


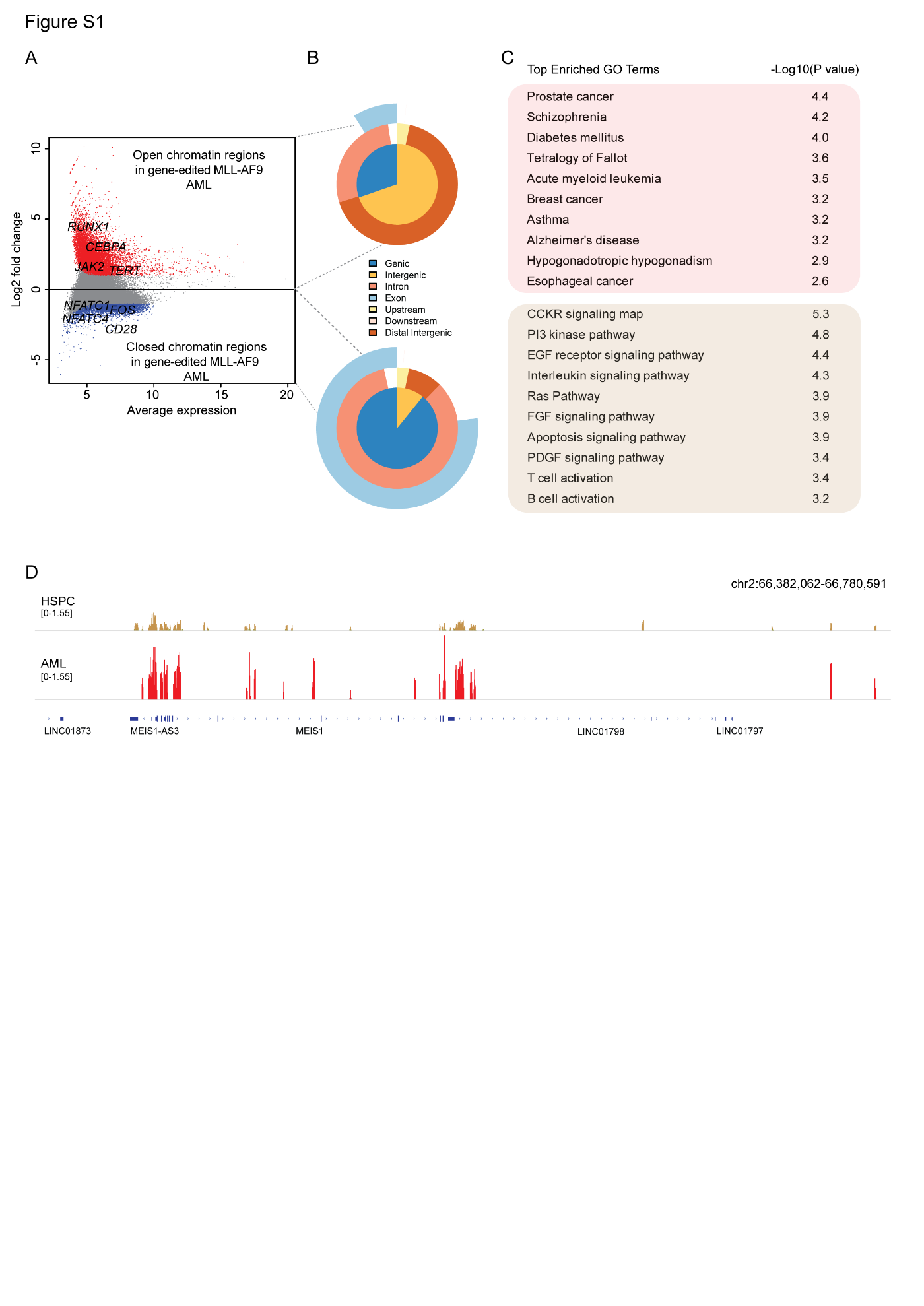


**Figure S1: Comparison of chromatin accessibility between HSPC and MLL-AF9 AML.**

(A) MA plot of the normalized ATAC-seq intensities of cell type-specific peaks from healthy donor and diseased individuals.

(B) Distribution of genomic features of differential regulatory elements.

(C) Top enriched Gene Ontology terms of differential peaks.

(D) Normalized ATAC-seq signal profiles at *MEIS1* locus in HSPC and MLL-AF9 AML.


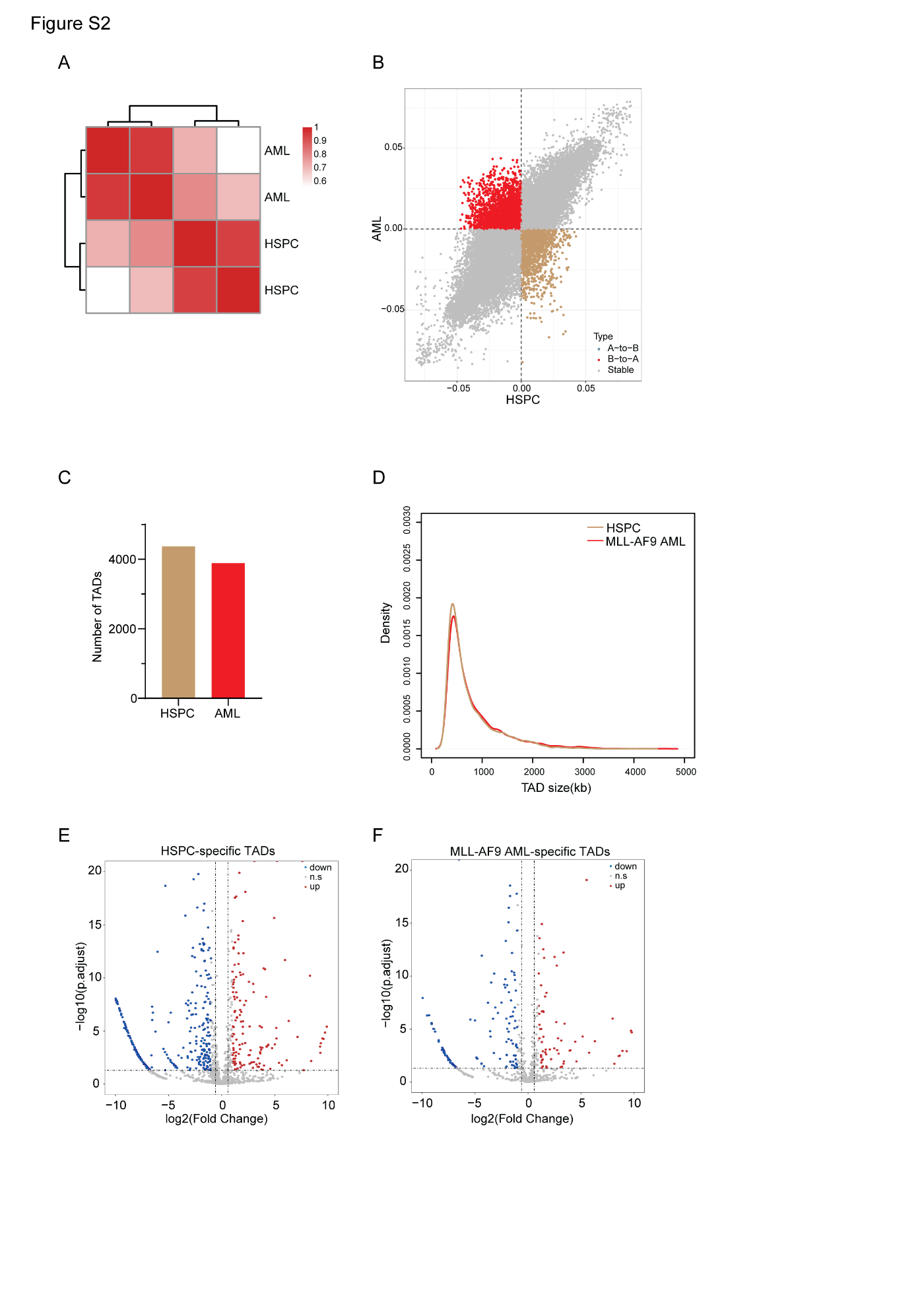


**Figure S2: A/B compartment switch and TAD alterations between HSPC and MLL-AF9 AML.**

(A) Unsupervised hierarchical clustering of the Micro-C signals between all the samples. Each row and each column is one sample.

(B) Dot plot of eigenvector shows the A/B compartment switch among HSPC and MLL-AF9 AML.

(C) Number of TADs in HSPC and MLL-AF9 AML.

(D) Size distribution of TADs of HSPC and MLL-AF9 AML at 25 kb resolution.

(E) Differential expression analysis of genes located inside HSPC-specific TADs.

(F) Differential expression analysis of genes located inside MLL-AF9 AML-specific TADs.


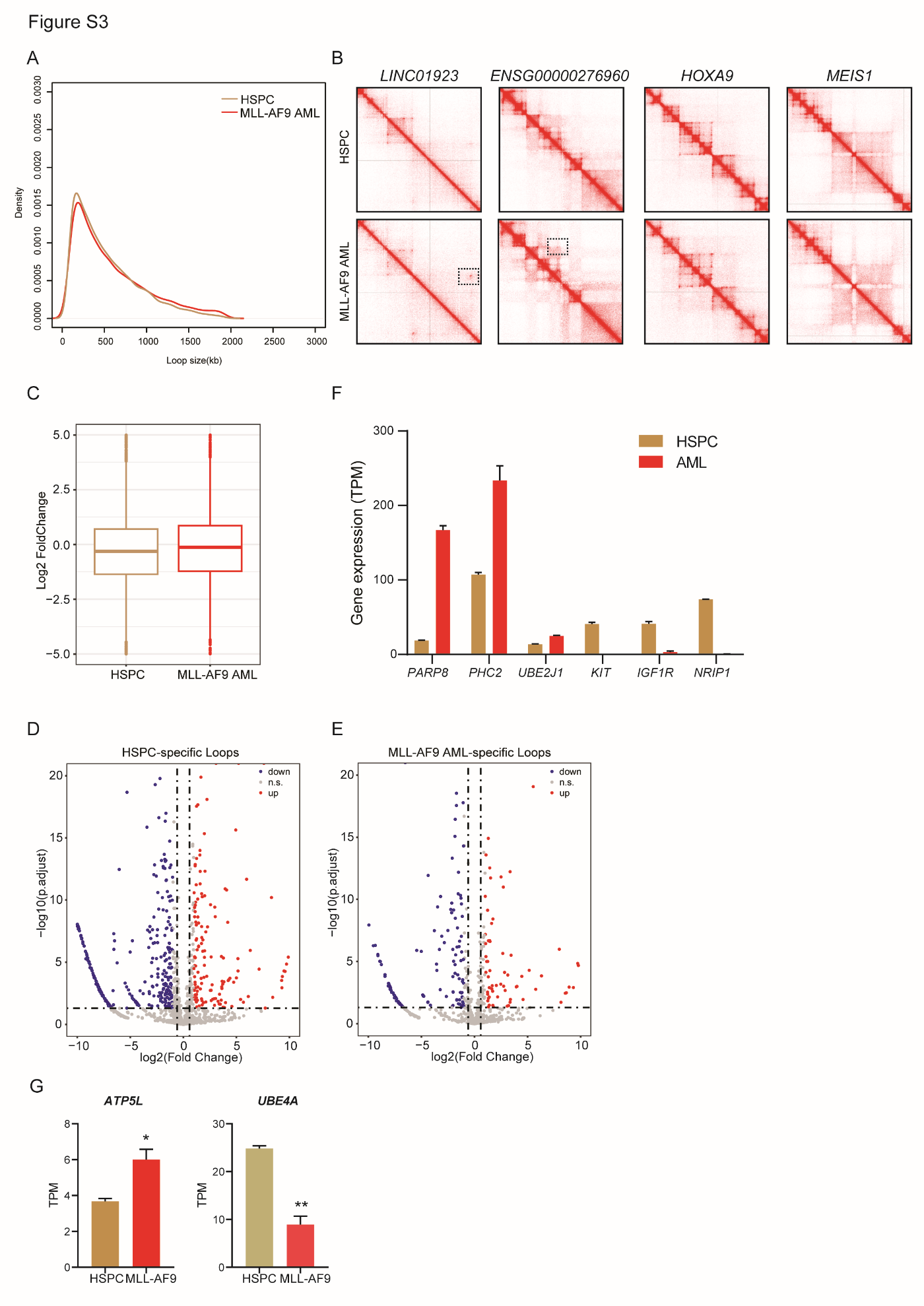


**Figure S3: MLL-AF9 AML-specific loop analysis.**

(A) Size distribution of loops of HSPC and MLL-AF9 AML at 10 kb resolution.

(B) Micro-C matrix surrounding AML noncoding elements (LINC01923 and ENSG00000276960) and MLLr signature genes (*HOXA9* and *MEIS1*).

(C) Gene expression alterations associated with loop changes.

(D) Differential expression analysis for genes in HSPC-specific loops.

(E) Differential expression analysis for genes in MLL-AF9 AML-specific loops.

(F) The gene expression (TPM) of HSPC and MLL-AF9 AML specific genes.

(G) The gene expression (TPM) of *ATP5L* and *UBE4A* in HSPC and MLL-AF9 AML. ∗P < .05; ∗∗P < .01.

**Reference**

1. Jeong, J., et al., *High-efficiency CRISPR induction of t(9;11) chromosomal translocations and acute leukemias in human blood stem cells.* Blood Adv, 2019. **3**(19): p. 2825-2835.

2. Li, H. and R. Durbin, *Fast and accurate short read alignment with Burrows-Wheeler transform.* Bioinformatics, 2009. **25**(14): p. 1754-60.

3. Open2C, et al., *Pairtools: from sequencing data to chromosome contacts.* bioRxiv, 2023.

4. Durand, N.C., et al., *Juicer Provides a One-Click System for Analyzing Loop-Resolution Hi-C Experiments.* Cell Syst, 2016. **3**(1): p. 95-8.

5. Abdennur, N. and L.A. Mirny, *Cooler: scalable storage for Hi-C data and other genomically labeled arrays.* Bioinformatics, 2020. **36**(1): p. 311-316.

6. Kruse, K., C.B. Hug, and J.M. Vaquerizas, *FAN-C: a feature-rich framework for the analysis and visualisation of chromosome conformation capture data.* Genome Biol, 2020. **21**(1): p. 303.

7. Roayaei Ardakany, A., et al., *Mustache: multi-scale detection of chromatin loops from Hi-C and Micro-C maps using scale-space representation.* Genome Biol, 2020. **21**(1): p. 256.

8. Flyamer, I.M., R.S. Illingworth, and W.A. Bickmore, *Coolpup.py: versatile pile-up analysis of Hi-C data.* Bioinformatics, 2020. **36**(10): p. 2980-2985.

9. Buenrostro, J.D., et al., *Transposition of native chromatin for fast and sensitive epigenomic profiling of open chromatin, DNA-binding proteins and nucleosome position.* Nat Methods, 2013. **10**(12): p. 1213-8.

10. Buenrostro, J.D., et al., *ATAC-seq: A Method for Assaying Chromatin Accessibility Genome-Wide.* Curr Protoc Mol Biol, 2015. **109**: p. 21 29 1-21 29 9.

11. Langmead, B. and S.L. Salzberg, *Fast gapped-read alignment with Bowtie 2.* Nat Methods, 2012. **9**(4): p. 357-9.

12. Bentsen, M., et al., *ATAC-seq footprinting unravels kinetics of transcription factor binding during zygotic genome activation.* Nat Commun, 2020. **11**(1): p. 4267.

13. Dobin, A., et al., *STAR: ultrafast universal RNA-seq aligner.* Bioinformatics, 2013. **29**(1): p. 15-21.

14. Putri, G.H., et al., *Analysing high-throughput sequencing data in Python with HTSeq 2.0.* Bioinformatics, 2022. **38**(10): p. 2943-2945.

15. Love, M.I., W. Huber, and S. Anders, *Moderated estimation of fold change and dispersion for RNA-seq data with DESeq2.* Genome Biol, 2014. **15**(12): p. 550.
